# Protective role of frizzled-related protein B on matrix metalloproteinase induction in mouse chondrocytes

**DOI:** 10.1186/ar4599

**Published:** 2014-07-01

**Authors:** Carole Bougault, Sabrina Priam, Xavier Houard, Audrey Pigenet, Laure Sudre, Rik J Lories, Claire Jacques, Francis Berenbaum

**Affiliations:** 1 INSERM UMRS 938, UPMC, Univ Paris 06, Faculté de Médecine Pierre & Marie Curie Paris VI, Hôpital Saint-Antoine, AP-HP, 184 rue du Faubourg Saint-Antoine, Paris 75012, France; 2 Laboratory of Tissue Homeostasis and Disease, Skeletal Biology and Engineering Research Center, Department of Development and Regeneration, KU Leuven, Herestraat 49, Leuven B3000, Belgium; 3 Department of Rheumatology, Assistance Publique – Hôpitaux de Paris, Saint-Antoine Hospital, 184 rue du Faubourg Saint-Antoine, Paris 75012, France; 4 Inflammation–Immunopathology–Biotherapy Department (DHU i2B), 184 rue du Faubourg Saint-Antoine, Paris 75012, France

## Abstract

**Introduction:**

Our objective was to investigate whether a lack of frizzled-related protein B (FrzB), an extracellular antagonist of the Wnt signaling pathways, could enhance cartilage degradation by facilitating the expression, release and activation of matrix metalloproteinases (MMPs) by chondrocytes in response to tissue-damaging stimuli.

**Methods:**

Cartilage explants from FrzB^−/−^ and wild-type mice were challenged by excessive dynamic compression (0.5 Hz and 1 MPa for 6 hours). Load-induced glycosaminoglycan (GAG) release and MMP enzymatic activity were assessed. Interleukin-1β (IL-1β) (10, 100 and 1000 pg/mL for 24 hours) was used to stimulate primary cultures of articular chondrocytes from FrzB^−/−^ and wild-type mice. The expression and release of MMP-3 and −13 were determined by RT-PCR, western blot and ELISA. The accumulation of β-catenin was assessed by RT-PCR and western blot.

**Results:**

Cartilage degradation, as revealed by a significant increase in GAG release (2.8-fold, *P* = 0.014) and MMP activity (4.5-fold, *P* = 0.014) by explants, was induced by an excessive load. Load-induced MMP activity appeared to be enhanced in FrzB^−/−^ cartilage explants compared to wild-type (*P* = 0.17). IL-1β dose-dependently induced *Mmp-13* and −*3* gene expression and protein release by cultured chondrocytes. IL-1β-mediated increase in MMP-13 and −3 was slightly enhanced in FrzB^−/−^ chondrocytes compared to wild-type (*P* = 0.05 and *P* = 0.10 at gene level, *P* = 0.17 and *P* = 0.10 at protein level, respectively). Analysis of *Ctnn1b* and *Lef1* gene expression and β-catenin accumulation at protein level suggests that the enhanced catabolic response of FrzB^−/−^ chondrocytes to IL-1β and load may be associated with an over-stimulation of the canonical Wnt/β-catenin pathway.

**Conclusions:**

Our results suggest that FrzB may have a protective role on cartilage degradation and MMP induction in mouse chondrocytes by attenuating deleterious effects of the activation of the canonical Wnt/β-catenin pathway.

## Introduction

Osteoarthritis (OA) is a common multifactorial joint disease, with ageing and excessive loading as important risk factors. Although the pathophysiology of OA involves cartilage, bone and the synovial tissue, the main feature of OA remains the progressive degradation of articular cartilage. Progressive joint destruction in OA has been associated with overactivation of Wnt signaling in numerous studies [1-6]. The Wnt signaling pathway is a potent regulator of bone and cartilage homeostasis and also has a role in human joint diseases [7,8]. The canonical Wnt pathway is initiated by binding of Wnt ligands to frizzled receptors and co-receptors, low-density lipoprotein receptors (LRP-5/6), which leads to intracellular β-catenin stabilization and accumulation, nuclear translocation, interaction with transcription factors, T-cell factor and lymphoid enhancer binding factor (LEF), and, finally, activation of target genes. The noncanonical Wnt pathways involve specific ligands and are independent of β-catenin and LRPs. Wnt ligands such as Wnt-7b, Wnt-16 and the Wnt target gene Wnt-1 inducible signaling pathway protein 1 (Wisp-1) were found upregulated in OA cartilage [1-3]. In addition, β-catenin, the co-receptor LRP-5 and the transcription factor LEF-1 were found overexpressed [4-6]. Frizzled-related protein B (FrzB) is an extracellular antagonist of the Wnt signaling pathway, also called secreted Frizzled-related protein 3 (sFRP-3). As FrzB can bind Wnts in the extracellular space and prevent ligand–receptor interaction, the protein can be considered an antagonist of both canonical and noncanonical signaling. Two single-nucleotide polymorphisms in *FRZB*, which are loss-of-function mutations, were associated with an increased risk of OA [9-13]. However, this association was challenged by recent studies [14-17], so the potential role of FrzB in OA is still controversial.

Studies in mouse models of OA corroborated the association between cartilage degradation and an overactivation of Wnt signaling. In particular, Wisp-1 was found upregulated in two models of OA; moreover, local overexpression of Wisp-1 enhanced cartilage damage [3]. Transgenic mice that produced activated-β-catenin in adult chondrocytes developed an OA-like phenotype upon ageing [4]. FrzB^−/−^ knockout mice did not develop spontaneous OA, but the deletion of *FrzB* increased cartilage loss in three different models of arthritis [18]. FrzB may thus have a protective role on OA progression. How FrzB can influence OA process remains largely unclear, but various hypotheses have been suggested [4,18-22]. The canonical Wnt pathway is crucial for proper chondrocyte differentiation in early developmental processes to control chondrogenesis and, later, to regulate hypertrophic maturation. Abnormal Wnt signaling in the absence of FrzB could cause aberrant skeletal morphogenesis, and variations in human hip shape have been associated with the abovementioned FrzB polymorphisms [19]. This signaling could contribute to the development of OA by increasing the biomechanical burden on the articular cartilage [19,23]. In addition, OA is also characterized by hypertrophy-like changes in chondrocytes, which could be enhanced by an overactivation of Wnt signaling in absence of FrzB [24].

In cultured chondrocytes, Wnt-3a – a commonly used Wnt ligand that triggers β-catenin signaling – increased the expression of matrix metalloproteinase (MMP)-3 and MMP-13, and MMP-2 and MMP-9 enzymatic activities [25-27]. In transgenic mice, activated β-catenin increases the expression of MMP-2, MMP-3, MMP-7, MMP-9 and MMP-13 [4,28]. Similarly, downregulation of LRP-5 decreased the expression of MMP-7, MMP-9, MMP-13 and MMP-14 [5,6]. The transcription complex formed by activated-β-catenin and Lef-1 has been shown to strongly bind MMP-9, MMP-13 and MMP-14 promoters, especially [5,29].

We focused on the hypothesis that the absence of FrzB could favor OA-like catabolic processes in cartilage by increasing the activation of the Wnt signaling pathway. We therefore studied cartilage degradation in FrzB KO mice, after biomechanical loading or cytokine treatment.

## Methods

### Animals

All experiments were made on explants or primary chondrocytes extracted from 3-day-old to 6-day-old newborn litters from FrzB^*−/−*^ or wild-type mice [18]. All procedures were in accordance with the European Directive N886/609 and were performed according to the protocols approved by French and European ethics committees for animal use and care (Comité Régional d’Ethique en Expérimentation Animale N°3 de la région Ile de France).

### Compression of costal cartilage explants

The procedure for compressive loading of mouse costal cartilage explants was as described previously [30]. Briefly, explants were harvested from rib cages of 4-day-old to 6-day-old newborn mice. Samples were cleaned, divided into segments, pooled and weighed for further normalization; each sample consisted of around 30 to 40 mg costal cartilage. The explants were allowed to rest for about 20 hours in 3 ml serum-free medium (Dulbecco’s modified Eagle’s medium supplemented with 0.1% bovine serum albumin and 30 mM Hepes). They were washed before they underwent 6-hour dynamic compression in 1.5 ml of the same fresh medium (sinusoidal wave-form 0 to 1 MPa at 0.5 Hz) by the Biopress system (Flexercell International, Dunn Labortechnick GmbH, Asbach, Germany). Control explants were kept in unloaded conditions. After the application of the mechanical regimen, supernatants and cartilage explants were collected and stored immediately at −80°C. Our results are expressed as fold-induction versus the uncompressed explant controls.

### Primary culture of mouse articular chondrocytes

Primary chondrocytes were isolated from articular cartilage of 4-day-old to 6-day-old newborn mice as described previously [31]. After 1 week of expansion, the cells were placed in serum-free conditions for 24 hours (0.1% bovine serum albumin; Sigma Aldrich, Saint-Quentin Fallavier, France) and then treated for 24 hours in serum-free medium supplemented with 0, 10, 100 or 1,000 pg/ml interleukin (IL)-1β (PeproTech, Tebu-Bio, Le Perray-en-Yvelines, France). Our results are expressed as fold-induction versus the nontreated controls.

### RNA extraction, reverse transcription and real-time polymerase chain reaction

RNA was extracted from chondrocytes cultured with and without IL-1β by use of the RNeasy minikit (Qiagen, Courtaboeuf, France); for the RNA extraction from compressed and uncompressed cartilage explants, Proteinase K (Qiagen) was first added to remove proteins as suggested for tissue samples by the manufacturer. Reverse transcription was performed on 1 μg RNA for monolayer cultured cells and on 100 to 200 ng RNA for explants using the Omniscript kit (Qiagen). Relative quantification of genes involved use of the LC480 LightCycler Real Time PCR system (Roche Applied Science, Meylan, France) and the Go Taq QPCR master mix (Promega, Charbonnières les Bains, France). mRNA levels of MMP-3 and MMP-13, β-catenin (*Ctnnb1*) and Lef1 were normalized to that of hypoxanthine–guanine phosphoribosyltransferase (*Hprt*), used as the internal standard.

The primes used (sense and antisense) were as follows: *Mmp-3*, s-TGAAAATGAAGGGTCTTCCGG and as-GCAGAAGCTCCATACCAGCA; *Mmp-13*, s-GATGGCACTGCTGACATCAT and as-TGTAGCCTTTGGAACTGCTT; *Ctnnb1*, s-GCAGCAGCAGTTTGTGGA and as-TGTGGAGAGCTCCAGTACACC; *Lef1*, s-TCCTGAAATCCCCACCTTCT and as-TGGGATAAACAGGCTGACCT; and *Hprt*, s-AGGACCTCTCGAAGTGT and as-ATTCAAATCCCTGAAGTACTCAT.

### MMP-3 and MMP-13 protein measurement

The amount of MMP-3 and MMP-13 released by chondrocytes in response to IL-1β was assessed in the culture supernatants. For total mouse MMP-3 secretion we used a commercially available enzyme-linked immunosorbent assay kit (R&D Systems, Lille, France). For total mouse MMP-13 secretion we performed western blot analysis as described previously [32] with rabbit polyclonal antibody for MMP-13 (H-230; Santa Cruz Biotechnology, Tebu-Bio). Densitometry analysis of immunoblots involved use of Multi Gauge software (Fujifilm, Paris, France).

### Glycosaminoglycan and matrix metalloproteinase enzymatic activity assays

The amount of glycosaminoglycan and global MMP activity was measured in the culture supernatants of compressed cartilage explants. Released glycosaminoglycan was assessed by reaction with dimethylmethylene blue [33]. Shark chondroitin sulfate was used as a standard. MMP activity was assessed by Mca-Pro-Leu-Gly-Leu-Dpa-Ala-Arg-NH_2_ synthetic fluorogenic substrate (Bachem, Weil am Rhein, Germany) in continuous assays [34]. Results were normalized to milligram wet weight cartilage and concentration of proteins in the culture supernatant.

### β-Catenin accumulation

Intracellular proteins were extracted from chondrocytes cultured with and without IL-1β. Assay of total mouse β-catenin involved western blot analysis with rabbit polyclonal antibody for β-catenin (Cell Signaling, Danvers, MA, USA). A reprobing with anti-β-actin antibodies (AC-15; Sigma Aldrich) served as a loading control. Densitometry analysis of immunoblots involved use of Multi Gauge software (Fujifilm). The ratio of β-catenin band intensity to the β-actin band intensity was calculated.

### Statistical analysis

Data are expressed as the mean ± standard error of the mean and were analyzed by the use of Mann–Whitney nonparametric tests. Three to four independent experiments were performed. *P* values and number of experiments (*n*) are indicated in the figure legends.

## Results

### Load-induced catabolism is enhanced in FrzB^−/−^ cartilage

**Figure 1:**
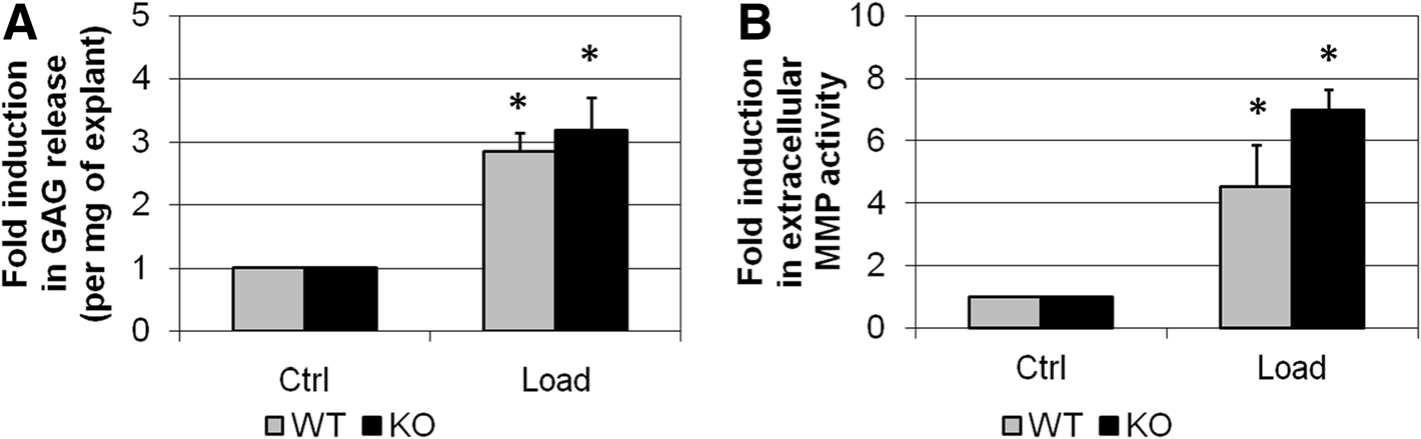
**Load-induced glycosaminoglycan release and matrix metalloproteinase activity in mouse cartilage explants from FrzB**^**−/− **^**and wild-type mice.** Explants were subjected to dynamic compression for 6 hours (0.5 Hz, 1 MPa). FrzB^−/−^ cartilage explants (black bars, *n* = 4) or wild-type (WT) explants (gray bars, *n* = 4) were loaded. Results from the loaded cartilage explants were normalized to those from the corresponding nonloaded explants (Ctrl), so that the graphs represent the fold-induction in response to compression. **(A)** Amount of glycosaminoglycan (GAG) released from cartilage explants into culture supernatant. **(B)** Matrix metalloproteinase (MMP) enzymatic activity was measured in the culture supernatant of cartilage explants. Load-induced MMP activity tends to be enhanced in FrzB^−/−^ explants compared with WT (*P* = 0.17). Bars represent the mean ± standard error of the mean. *P ≤ 0.05 versus Ctrl. FrzB, frizzled-related protein B; KO, knockout.

Cartilage explants from FrzB^*−/−*^ and wild-type mice were stimulated by dynamic compression for 6 hours. As previously published by our group, load induced cartilage degradation in the explants [35]. Cartilage degradation was revealed by a net increase in glycosaminoglycan release (2.8-fold, *P* = 0.014; Figure 1A) and MMP activity (4.5-fold, *P* = 0.014; Figure 1B). At baseline, cartilage degradation was not different between the FrzB^−/−^ and wild-type samples (data not shown). Load-induced glycosaminoglycan release was similar in FrzB^−/−^ and wild-type samples but load-induced increase in MMP activity was slightly enhanced in FrzB^−/−^ cartilage explants compared with wild-type explants (1.6-fold, *P* = 0.17; Figure 1B). The lack of FrzB thus tends to enhance the catabolic response of chondrocytes *in situ*, in response to mechanical stress.

### IL-1β-mediated MMP induction is enhanced in FrzB^−/−^ chondrocytes

**Figure 2:**
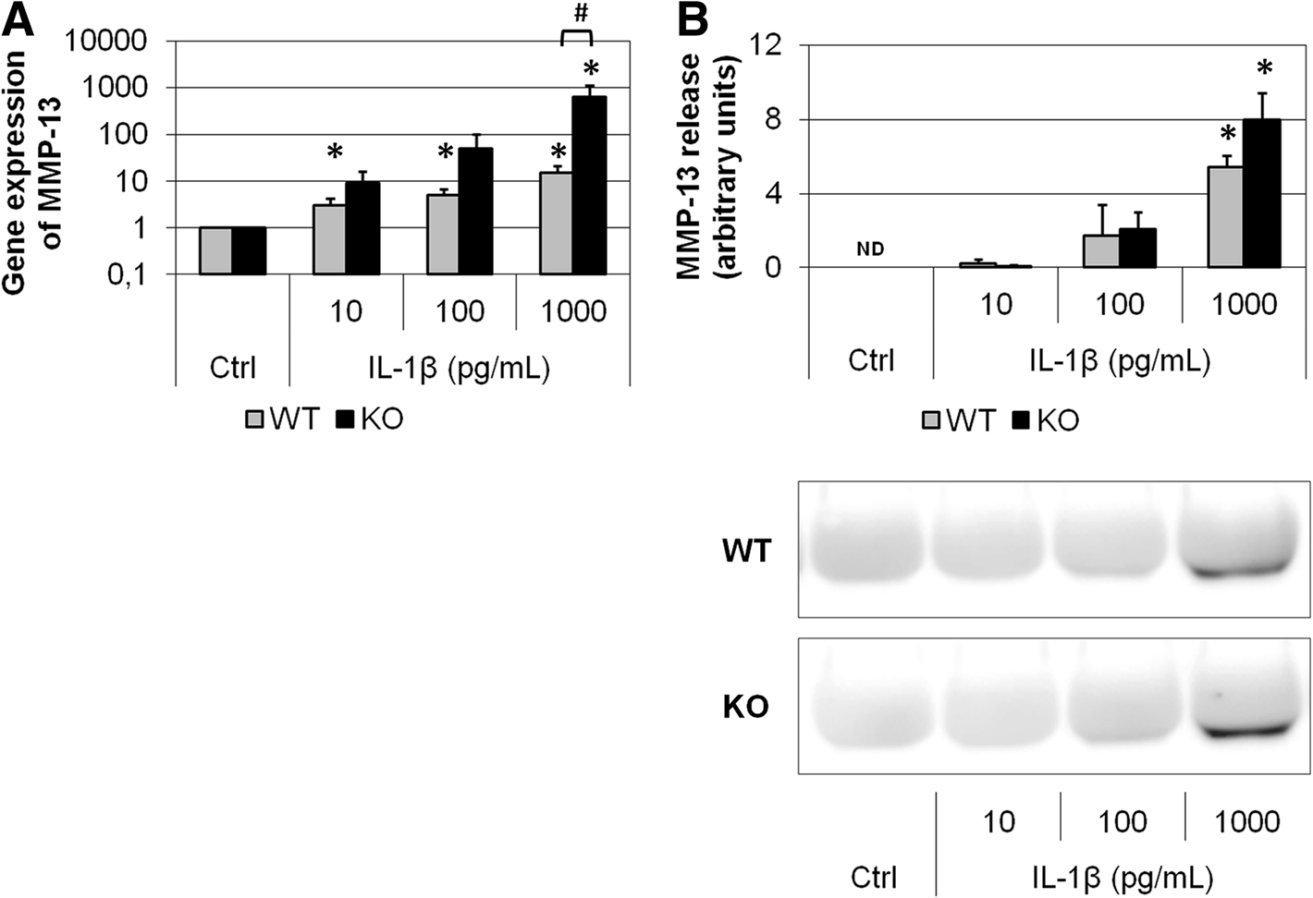
**IL-1β-mediated increase in MMP-13 in cultured articular chondrocytes is enhanced in absence of FrzB.** Primary chondrocytes were treated for 24 hours with interleukin (IL)-1β (10, 100 or 1,000 pg/ml). Results from the treated chondrocytes were normalized to those from nontreated samples (Ctrl), so that the graphs represent the fold-induction in response to IL-1β. **(A)** Real-time polymerase chain reaction analysis of *Mmp-13* gene expression. IL-1β-induced *Mmp-13* gene expression tends to be enhanced in FrzB^−/−^ chondrocytes compared with wild-type (WT) chondrocytes, especially with 1,000 pg/ml (*P* = 0.05, n = 3). **(B)** Culture media were analyzed for MMP-13 by western blotting. Quantification of the MMP-13 blot is shown above the blot. IL-1β-induced MMP-13 protein release tends to be enhanced in FrzB^−/−^ chondrocytes compared with WT chondrocytes, especially with 1,000 pg/ml (*P* = 0.17, *n* = 4). Bars represent the mean ± standard error of the mean. *P ≤ 0.05 versus Ctrl, #*P* ≤ 0.10 between FrzB^−/−^ and WT. FrzB, frizzled-related protein B; KO, knockout; MMP, matrix metalloproteinase; ND, not detected.

**Figure 3:**
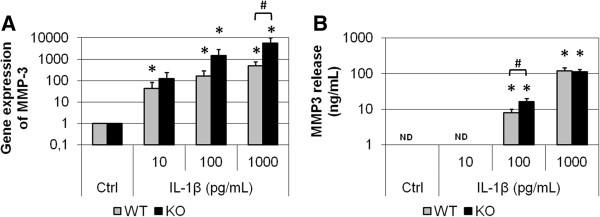
**IL-1β-mediated increase in MMP-3 in cultured articular chondrocytes is enhanced in absence of FrzB.** Primary chondrocytes were treated for 24 hours with interleukin (IL)-1β (10, 100 or 1,000 pg/ml). **(A)** Real-time polymerase chain reaction analysis of *Mmp-3* gene expression. Results from the treated chondrocytes were normalized to those from nontreated samples (Ctrl), so that the graphs represent the fold-induction in response to IL-1β. The IL-1β-induced *Mmp-3* gene expression tends to be enhanced in FrzB^−/−^ chondrocytes compared with wild-type (WT) chondrocytes, especially with 1,000 pg/ml (*P* = 0.10, *n* = 3). **(B)** Culture media were analyzed for total MMP-3 by enzyme-linked immunosorbent assay. MMP-3 protein release in response to 100 pg/ml IL-1β tends to be enhanced in FrzB^−/−^ chondrocytes compared with WT chondrocytes (*P* = 0.10, *n* = 3). Bars represent the mean ± standard error of the mean. *P ≤ 0.05 versus Ctrl, #*P* ≤ 0.10 between FrzB^−/−^ and WT. FrzB, frizzled-related protein B; KO, knockout; MMP, matrix metalloproteinase; ND, not detected.

Primary cultures of articular chondrocytes from FrzB^−/−^ and wild-type mice were stimulated with increasing doses of IL-1β for 24 hours (10, 100 and 1000 pg/ml). IL-1β dose-dependently induced *Mmp-13* gene expression (up to 15-fold, *P* = 0.05; Figure 2A). At the higher dose, IL-1β-induced *Mmp-13* gene expression was enhanced in FrzB^−/−^ chondrocytes compared with wild-type chondrocytes (*P* = 0.05). Of note, in basal conditions *Mmp-13* gene expression was similar in FrzB^−/−^ and wild-type chondrocytes (0.012 ± 0.008 vs. 0.020 ± 0.008 arbitrary units, respectively). At protein level, MMP-13 release was measured by western blot analysis (Figure 2B). MMP-13 release was not modulated by the lower doses of IL-1β, but was significantly increased in response to 1,000 pg/ml (*P* = 0.014). This increase in MMP-13 release tended to be slightly enhanced in FrzB^−/−^ chondrocytes compared with wild-type chondrocytes (1.5-fold, *P* = 0.17). Similarly, IL-1β dose-dependently induced *Mmp-3* gene expression (up to 500-fold, *P* = 0.05; Figure 3A). At the higher dose, IL-1β-induced *Mmp-3* gene expression tended to be enhanced in FrzB^−/−^ chondrocytes compared with wild-type chondrocytes (*P* = 0.10). As for *Mmp-13*, *Mmp-3* gene expression was similar in FrzB^−/−^ and wild-type chondrocytes in basal conditions (0.0021 ± 0.0010 vs. 0.0017 ± 0.0004 arbitrary units, respectively). At protein level, MMP-3 release was assessed by enzyme-linked immunosorbent assay (Figure 3B). MMP-3 was not detected in untreated conditions, but only in response to the higher doses of IL-1β (*P* = 0.05). MMP-3 release in response to 100 pg/ml IL-1β was enhanced in FrzB^−/−^ chondrocytes compared with wild-type (2.1-fold, *P* = 0.10). The absence of FrzB thus tends to enhance the catabolic response of cultured chondrocytes, in response to a proinflammatory stress. These results are parallel to the enhanced catabolic response observed in loaded FrzB^−/−^ cartilage explants.

### IL-1β-induced and load-induced catabolism in FrzB^−/−^ chondrocytes is associated with canonical Wnt/β-catenin signaling

We wondered whether the enhancement in the response of FrzB^−/−^ chondrocytes to IL-1β and load was associated with a deregulation in Wnt/β-catenin signaling. In basal conditions, *Ctnnb1* gene expression (coding β-catenin) was comparable between FrzB^−/−^ cultured chondrocytes and wild-type chondrocytes (0.62 ± 0.22 vs. 0.95 ± 0.12 arbitrary units, respectively). Of note, similar results were observed for the Wnt/β-catenin target gene *Lef1* (0.031 ± 0.009 for wild-type chondrocytes vs. 0.018 ± 0.013 for FrzB^−/−^ chondrocytes). These data are in accordance with the previous transcriptome analysis of the bone–cartilage unit of FrzB^−/−^ mice: at baseline *in vivo*, the *Ctnnb1* mRNA level was not different between the FrzB^−/−^ and wild-type strains [36].

**Figure 4:**
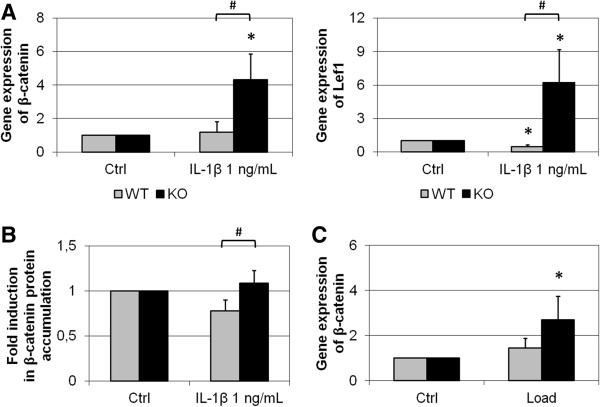
**IL-1β-mediated and load-mediated regulation of Wnt/β-catenin signaling in chondrocytes from FrzB**^**−/− **^**and wild-type mice.** Cultured articular chondrocytes from FrzB^−/−^ mice or wild-type (WT) mice were treated for 24 hours with interleukin (IL)-1β (1 ng/ml). Results from the IL-1β-treated samples were normalized to the control ones (Ctrl), so that the graphs represent the fold-induction in response to IL-1β. **(A)***Ctnnb1* gene expression (coding β-catenin) and *Lef1* gene expression (a Wnt/β-catenin target gene) were analyzed by real-time polymerase chain reaction (PCR; *n* = 4 and *n* = 3, respectively). *Ctnnb1* gene expression was not modulated by IL-1β in WT chondrocytes and *Lef1* gene expression was decreased (*P* = 0.05). In contrast, the treatment induced *Ctnnb1* and *Lef1* gene expression in FrzB^−/−^ chondrocytes (*P* = 0.014 and *P* = 0.05, respectively). IL-1β-mediated regulation of β-catenin expression was thus different between FrzB^−/−^ and WT (*P* = 0.057 for *Ctnnb1*, *P* = 0.05 for *Lef1*). **(B)** Intracellular extracts were analyzed for total β-catenin by western blotting. Quantification of β-catenin blot suggested that IL-1β-mediated β-catenin accumulation was different between FrzB^−/−^ and WT (*P* = 0.10, *n* = 4). **(C)** FrzB^−/−^ cartilage explants or WT explants were subjected to dynamic compression for 6 hours (0.5 Hz, 1 MPa). *Ctnnb1* gene expression was analyzed by real-time PCR (*n* = 3). Results from the loaded cartilage explants were normalized to those from the corresponding nonloaded explants (Ctrl), so that the graphs represent the fold-induction in response to compression. *Ctnnb1* gene expression was not affected by load in WT explants but was increased 2.7 fold in compressed FrzB^−/−^ samples (*P* = 0.05). The load-induced increase in *Ctnnb1* mRNA tends to be enhanced in FrzB^−/−^ explants compared with WT explants (*P* = 0.20). Bars represent the mean ± standard error of the mean, *P ≤ 0.05 versus Ctrl, #*P* ≤ 0.10 between FrzB^−/−^ and WT. FrzB, frizzled-related protein B; KO, knockout.

*Ctnnb1* gene expression was not affected by IL-1β in wild-type cultured chondrocytes (Figure 4A). In IL-1β-treated FrzB^−/−^ chondrocytes, *Ctnnb1* gene expression was induced 4.3-fold compared with untreated chondrocytes (*P* = 0.014). *Ctnnb1* gene expression was thus markedly higher in IL-1β-treated FrzB^−/−^ chondrocytes compared with wild-type chondrocytes (3.6-fold, *P* = 0.057). In parallel, *Lef1* gene expression was induced 6.2-fold in IL-1β-treated FrzB^−/−^ chondrocytes compared with untreated chondrocytes (*P* = 0.05) and the *Lef1* mRNA level was clearly higher in IL-1β-treated FrzB^−/−^ chondrocytes compared with wild-type chondrocytes (13-fold, *P* = 0.05; Figure 4A). Analysis of β-catenin accumulation at the protein level did not strictly correlate this transcriptional regulation (Figure 4B). IL-1β tended to decrease the β-catenin content of wild-type cultured chondrocytes (20% less, *P* = 0.057), whereas no modulation was observed in FrzB^−/−^ chondrocytes. The β-catenin content was thus higher in IL-1β-treated FrzB^−/−^ chondrocytes compared with wild-type chondrocytes (1.4-fold, *P* = 0.10). IL-1β only slightly modulates Wnt/β-catenin signaling in our model, but FrzB-dependent deregulation was observed in IL-1β-treated chondrocytes.

Concerning the catabolic response of FrzB^−/−^ cartilage to load, analysis of *Ctnnb1* gene expression showed very similar results (Figure 4C). The *Ctnnb1* mRNA level was not affected by compression in wild-type explants. In compressed FrzB^−/−^ samples, *Ctnnb1* gene expression was induced 2.7-fold compared with uncompressed samples (*P* = 0.05). The modulation in Wnt/β-catenin signaling in response to load was thus slightly enhanced in FrzB^−/−^ cartilage (*P* = 0.20; Figure 4C).

Overall, these results suggest that the enhancement in the response of FrzB^−/−^ chondrocytes to IL-1β and load may be associated with an overstimulation of the canonical Wnt pathway.

## Discussion

### Enhanced responsiveness to mechanical stress in the absence of FrzB and involvement of the canonical Wnt/β-catenin pathway

We have demonstrated that load-induced MMP activity was enhanced in FrzB^−/−^ cartilage explants. In addition, load stimulated *Ctnnb1* gene expression in FrzB^−/−^ explants. These results suggest that the load-induced catabolic response of chondrocytes may be in part mediated by the canonical Wnt pathway. The involvement of Wnt signaling in osteoblast responsiveness to mechanical stimulation was proven in various studies [37-42]. In particular, TOPGAL reporter mice showed that the canonical Wnt/β-catenin pathway was activated by mechanical stress *in vivo* and in cultured osteoblasts [43]. Furthermore, increasing basal β-catenin levels was shown to enhance the effects of mechanical stress [44]. Chondrocyte responses towards mechanical stimulation have been less studied. However, pressure-induced mechanical stress triggered β-catenin tyrosine phosphorylation in cultured chondrocytes [45], thereby probably releasing the molecule from adherens junctions and increasing its availability for intracellular signaling. Partial β-catenin nuclear translocation was also observed in response to tensile strain [27]. Furthermore, there was an additive effect of load and Wnt-3a on β-catenin translocation and on upregulation of *Mmp-3* gene expression [27]. Of interest, a downregulation of FrzB was observed in human and mouse cartilage explants in response to mechanical injury, suggesting a de-repression of the canonical Wnt pathway [46]. Wnt/β-catenin signaling may thus be part of the signaling response leading to excessive catabolism and cartilage degradation in response to abnormal loading and FrzB may have a protective role on load-induced increase in MMP activity.

### Enhanced responsiveness to IL-1β in the absence of FrzB and involvement of the canonical Wnt/β-catenin pathway

We demonstrated that IL-1β-mediated increases in *Mmp-3* and *Mmp-13* gene expression and protein release were enhanced in FrzB^−/−^ chondrocytes. Similarly, IL-1β stimulated *Ctnnb1* and *Lef1* gene expression and β-catenin accumulation in FrzB^−/−^ chondrocytes. Canonical Wnt pathway activation may thus enhance chondrocyte responsiveness to IL-1β. These results suggest a crosstalk between the canonical Wnt pathway, which includes β-catenin and FrzB, and the IL-1 pathway, which stimulates MMP expression in chondrocytes. In accordance with our results, activation of β-catenin signaling in cultured chondrocytes by Wnt-3a treatment potentiated IL-1β-mediated loss of proteoglycans [25]. Conversely, inhibition of β-catenin signaling by the use of Lef1 siRNA downregulated IL-1β-mediated increase in *Mmp-13* gene expression [29]. Sost, a biologically active inhibitor of β-catenin signaling in chondrocytes, downregulated the IL-1α-mediated increase in *Mmp-13* gene expression in cartilage explants and also reduced the loss of proteoglycans [47]. An alternative suggestion is that IL-1β treatment may induce production of canonical Wnt such as Wnt-7b, which in turn would activate β-catenin signaling [48]. In surprising contrast, in human chondrocytes β-catenin signaling was found to counteract IL-1β-mediated increase in MMP-3 and MMP-13 expressions [48]. In conclusion, the canonical Wnt pathway may be part of mechanisms leading to excessive catabolism in response to inflammatory stress and FrzB may have a protective role on IL-1-mediated increase in MMP expression in mouse chondrocytes.

### Putative protective role of FrzB in osteoarthritis progression

In OA, cartilage breakdown is due to cleavage of matrix molecules in response to abnormal mechanical stress and to some degree of inflammation. Because our results suggest that FrzB may have a protective role on load-mediated and IL-1-mediated catabolic processes in mouse chondrocytes, we speculate that FrzB may have a protective role in OA. Our results are consistent with the increased cartilage loss observed in models of arthritis in FrzB^−/−^ knockout mice [18]. Moreover, gene expression of *Mmp-3* was upregulated in the cartilage of FrzB^−/−^ mice with mBSA-induced arthritis compared with wild-type mice. However, FrzB^−/−^ knockout mice did not develop spontaneous OA, and they did not show aberrant *Mmp* gene expressions in basal conditions, except for a twofold increase for MMP-3 [36]. FrzB may thus be involved in OA progression rather than OA onset. Although FrzB may interact directly with MMP-3 [18], its protective role is probably linked with a deregulation of the Wnt pathways. The recent identification of FrzB as a blocker of hypertrophic differentiation in articular cartilage [22] promotes the hypothesis of a protective role of FrzB in OA through the Wnt-mediated regulation of hypertrophic maturation.

## Conclusions

Our results suggest that FrzB has a protective role on MMP induction in mouse chondrocytes. The results indicate a dual role of Wnt signaling in cartilage homeostasis, so that a controlled amount of Wnt signaling is necessary for maintenance of the articular cartilage, but an excess one is deleterious. Further investigations are needed to decipher the tight control of Wnt signaling in OA, in particular concerning the differentiation of OA chondrocytes towards hypertrophy.

Our results also add evidence to demonstrate that the canonical Wnt/β-catenin pathway is part of mechanisms leading to excessive catabolism and cartilage degradation in OA. However, the FrzB-dependent deregulation that we observed may involve both canonical and noncanonical Wnt signaling since FrzB is an inhibitor of both pathways. Little is known concerning the role of the noncanonical Wnt pathway in articular cartilage homeostasis and OA development. Recent data suggested that, in excess, Wnt-5a could stimulate degradation of the mature cartilage matrix via noncanonical pathways, while promoting normal differentiation in developing cartilage [49]. Additional investigations on Wnt regulation in OA should therefore equally explore canonical and noncanonical Wnt pathways.

## Abbreviations

FrzB: frizzled-related protein B (secreted frizzled-related protein 3); IL: interleukin; LEF: lymphoid enhancer binding factor; LRP: low-density lipoprotein receptor; MMP: matrix metalloproteinase; OA: osteoarthritis.

## Competing interests

The authors declare that they have no competing interests.

## Authors’ contributions

CB, SP, XH, CJ and FB were responsible for conception and design. CB, SP, AP and LS were responsible for acquisition of data. CB, SP, XH, RJL, CJ and FB were responsible for analysis and interpretation of the data. CB, SP and AP were responsible for drafting of the article. XH, RJL, CJ and FB were responsible for critical revision of the article for important intellectual content. All authors were responsible for final approval of the article.

## Authors’ information

This work was supported by a grant from Fondation pour la Recherche Médicale. CB was supported by a Fondation pour la Recherche Médicale postdoctoral fellowship and by French state funds managed by the Agence Nationale de la Recherche within the Investissements d’Avenir programme under reference ANR-11-IDEX-0004-02.
